# Lyophilization of Curcumin–Albumin Nanoplex with Sucrose as Cryoprotectant: Aqueous Reconstitution, Dissolution, Kinetic Solubility, and Physicochemical Stability

**DOI:** 10.3390/ijms231911731

**Published:** 2022-10-03

**Authors:** Angeline Chua, The-Thien Tran, Siyu Pu, Jin-Won Park, Kunn Hadinoto

**Affiliations:** 1 School of Chemistry Chemical Engineering and Biotechnology, Nanyang Technological University, Singapore 637459, Singapore; 2 School of Chemical and Biomolecular Engineering, Seoul University of Science and Technology, Seoul 01811, Korea

**Keywords:** curcumin nanoparticles, albumin nanoparticles, amorphous drugs, freeze drying, protein stability

## Abstract

An amorphous curcumin (CUR) and bovine serum albumin (BSA) nanoparticle complex (nanoplex) was previously developed as a promising anticancer nanotherapy. The CUR-BSA nanoplex had been characterized in its aqueous suspension form. The present work developed a dry-powder form of the CUR-BSA nanoplex by lyophilization using sucrose as a cryoprotectant. The cryoprotective activity of sucrose was examined at sucrose mass fractions of 33.33, 50.00, and 66.66% by evaluating the lyophilized nanoplex’s (1) aqueous reconstitution and (2) CUR dissolution and kinetic solubility. The physicochemical stabilizing effects of sucrose upon the nanoplex’s 30-day exposures to 40 °C and 75% relative humidity were examined from (i) aqueous reconstitution, (ii) CUR dissolution, (iii) CUR and BSA payloads, (iv) amorphous form stability, and (v) BSA’s structural integrity. The good cryoprotective activity of sucrose was evidenced by the preserved BSA’s integrity and good aqueous reconstitution, resulting in a fast CUR dissolution rate and a high kinetic solubility (≈5–9× thermodynamic solubility), similar to the nanoplex suspension. While the aqueous reconstitution, CUR dissolution, and amorphous form were minimally affected by the elevated heat and humidity exposures, the treated nanoplex exhibited a lower BSA payload (≈7–26% loss) and increased protein aggregation postexposure. The adverse effects on the BSA payload and aggregation were minimized at higher sucrose mass fractions.

## 1. Introduction

The chemopreventive, chemotherapeutic, and chemoprotective activities of curcumin (CUR)—a natural polyphenol extracted from turmeric plants—have been broadly demonstrated in preclinical studies [1,2]. Clinical applications of CUR-based anticancer therapy, however, have only found limited success due to CUR’s poor bioavailability caused by its low aqueous solubility [3,4]. CUR-loaded nanoparticles using polymers, lipids, and inorganic materials (e.g., mesoporous silica) as inert carriers represent one of the most effective formulation strategies to enhance CUR’s solubility due to the large specific surface areas afforded by nanoparticles [5,6,7,8]. Besides the solubility enhancement, CUR-loaded nanoparticles are particularly attractive for anticancer therapy because of the well-known enhanced permeation and retention (EPR) effects of nanoparticles, resulting in higher drug accumulation in the tumor sites [9]. 

Among the CUR nanoparticle formulations intended for anticancer therapy, CUR-loaded albumin nanoparticles stand out by virtue of albumin’s uniquely superior characteristics compared to other inert carriers. First, nanoparticles of albumin, as the most abundant protein in the plasma, inherently exhibit high biocompatibility, nontoxicity, minimal immunogenicity, and a long half-life [10]. Second, CUR readily binds to albumin via hydrophobic and/or electrostatic interactions, which facilitates highly efficient CUR incorporation into albumin nanoparticles [11]. Third, CUR-albumin interactions have been found to increase CUR’s aqueous solubility [12]. Fourth, and most importantly, albumin is known to have a high binding affinity to protein receptors overexpressed by tumor cells (e.g., gp60 and osteonectin). Therefore, using albumin as the carrier enables the active targeting of tumors without the need to equip the nanoparticles with targeting ligands [13].

In this regard, albumin nanoparticles containing the anticancer drug paclitaxel (Abraxane^®^) have been used clinically for more than a decade with well-demonstrated superior clinical outcomes to the conventional paclitaxel formulation [14]. Encouraged by the clinical success of Abraxane^®^, numerous studies on CUR-loaded albumin nanoparticles have been reported in recent years, where their anticancer activities were successfully demonstrated in both in vitro and in vivo studies [15]. The CUR-loaded albumin nanoparticles in the previous studies were predominantly prepared by desolvation (coacervation) using organic solvents as desolvating agents. This preparation method was similar to the nanoparticle albumin-bound (nab) technology employed in Abraxane^®^ preparation [11,16,17,18]. Crosslinking agents were sometimes added to strengthen the albumin nanoparticle structures [19].

In our previous study, we proposed an alternative method to prepare CUR-loaded albumin nanoparticles by an electrostatically and hydrophobically driven complexation between CUR and bovine serum albumin (BSA) [20]. Unlike the desolvation method [21], the CUR-BSA complexation method requires zero organic solvents and comprises only a single step of mixing ionized CUR and BSA solutions in ambient conditions. Besides its solvent-free and one-step features, the CUR-BSA complexation method produced albumin nanoparticles with higher CUR loading (>50 wt.%) than albumin nanoparticles prepared by desolvation, where the CUR loadings were typically below 20 wt.% [11,16,19]. Henceforth, the CUR-loaded albumin nanoparticles prepared by CUR-BSA complexation are referred as the CUR-BSA nanoparticle complex (or nanoplex for short).

The CUR-BSA nanoplex formation mechanisms were similar in principle to the mechanisms of the CUR-chitosan nanoplex formation described by Nguyen et al. [22]. Briefly, soluble CUR-BSA complexes were formed upon the mixing of the anionic CUR solution (pH > pK_a_) with the cationic BSA solution (pH < pI) as a result of the hydrophobic and electrostatic bindings between them. The soluble CUR-BSA complexes subsequently formed aggregates among themselves due to hydrophobic interactions among the bound CUR and BSA molecules. Upon reaching a critical particle mass, which was influenced by (i) the operating pH and (ii) the mass ratio of CUR to BSA, the CUR-BSA complex aggregates precipitated out of the solution to form an amorphous CUR-BSA nanoplex. The amorphous form of the CUR-BSA nanoplex resulted from the strong bindings between the CUR and BSA molecules, which in turn prevented both molecules from rearranging into ordered crystalline structures upon their precipitation.

The nanoscale size and the metastable amorphous form of the CUR-BSA nanoplex enabled it to generate a high kinetic solubility of CUR over a prolonged period (i.e., several hours) upon dissolution. The CUR kinetic solubility was multiple times higher than thermodynamic solubility of crystalline CUR [20]. Hitherto, the CUR solubility enhancement of the CUR-BSA nanoplex had only been evaluated in its aqueous suspension form. However, most pharmaceuticals typically have to undergo a drying process after their production to facilitate their storage in the dry-powder forms aimed at prolonging their shelf life [23]. The dry-powder transformation is particularly important for pharmaceutical proteins/peptides due to their low room-temperature stability [24]. Depending on the mode of administration (e.g., orally or parenterally) the dry-powder forms can be formulated into solid dosage forms (e.g., tablets) or reconstituted back into aqueous solution or suspension prior to their administration [25].

Lyophilization with common cryoprotectants (e.g., sucrose, mannitol, and trehalose) represents the most commonly used drying method for protein nanoparticles [26]. This is because high-temperature drying (e.g., spray drying) is not suitable for thermally labile compounds, such as albumin, which denatures at temperatures above 65 °C [27]. Besides protecting the protein structures from the stresses exerted by ice crystal formation during freezing, the cryoprotectant also plays an important role in preventing the irreversible aggregation of nanoparticles due to interparticle fusion, which in turn could adversely affect the nanoparticles’ therapeutic functions [28]. Significantly, for a given type of protein, the characteristics of the lyophilized nanoparticles are greatly influenced by the type of cryoprotectant used and its mass fraction in the final product [29,30,31].

In the present work, we aimed to evaluate the feasibility of using sucrose as the cryoprotectant for the lyophilization of the amorphous CUR-BSA nanoplex. Sucrose was selected as the cryoprotectant because its stabilization of free albumin protein during lyophilization had been previously reported [32]. The stabilization of BSA in the nanoplex during lyophilization was crucial because destabilized BSA would lead to adverse effects on the CUR-BSA nanoplex’s properties, such as colloidal stability, dissolution profiles, and the loss of BSA’s targeting ability. The cryoprotective activity of sucrose was evaluated based on the lyophilized nanoplex’s (1) aqueous reconstitution, (2) CUR dissolution profiles, and (3) amorphous CUR kinetic solubility. The investigations were carried out at different sucrose to nanoplex mass ratios in the range of 0 to 66.66% sucrose mass fractions.

Upon establishing sucrose as a feasible cryoprotectant for the CUR-BSA nanoplex, the present work proceeded to examine the ability of sucrose to preserve the physicochemical characteristics of the lyophilized nanoplex upon their 30-day exposures to elevated temperature (40 °C) and relative humidity (RH, 75%). In this regard, exposures to elevated temperature and humidity, which are often experienced by pharmaceutical products in their shelf life, are known to increase the recrystallization propensity of amorphous drugs [33] and the unfolding tendency of proteins, leading to protein aggregation [34]. The following properties of the lyophilized nanoplex were examined after the 30-day exposures: (1) the amorphous form stability, (2) the aqueous reconstitution, (3) the percentage reductions in the CUR and BSA payloads, (4) the CUR dissolution profiles, and finally (5) BSA’s structural integrity, as characterized by gel electrophoresis. 

## 2. Results and Discussion

### 2.1. Physical Characteristics of the Lyophilized CUR-BSA Nanoplex

**Figure 1:**
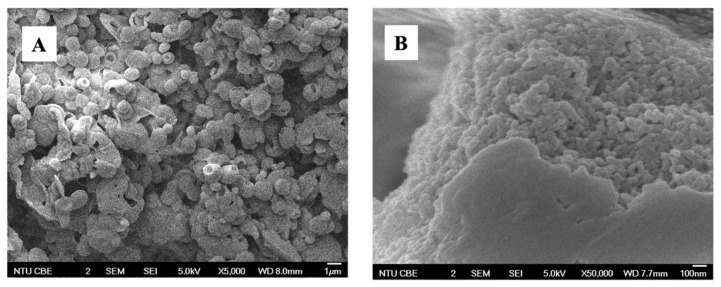
FESEM images of the lyophilized CUR-BSA nanoplex viewed at (**A**) low and (**B**) high magnifications.

Before lyophilization, the CUR-BSA nanoplex in its aqueous suspension form exhibited a size and zeta potential of 109 ± 26 nm ($S_{i}$) and −25.4 ± 1.8 mV, respectively, with a PDI of 0.29 ± 0.02. The negative zeta potential signified the presence of BSA in the vicinity of the nanoplex surface, as BSA carried a negative charge at a neutral pH because it was above its pI (4.5–5.0). Thus, BSA acted as colloidal stabilizer of the nanoplex. The FESEM analysis showed that the lyophilization of the CUR-BSA nanoplex with sucrose as the cryoprotectant resulted in roughly spherical particles with individual sizes between 1 and 2 µm (Figure 1A). A close-up view of the particles with exposed interiors confirmed the presence of the CUR-BSA nanoplex in the sucrose matrix (Figure 1B). Lyophilized nanoplex prepared at a 50.00% (*w*/*w*) sucrose mass fraction was used as the representative sample for FESEM. 

**Figure 2:**
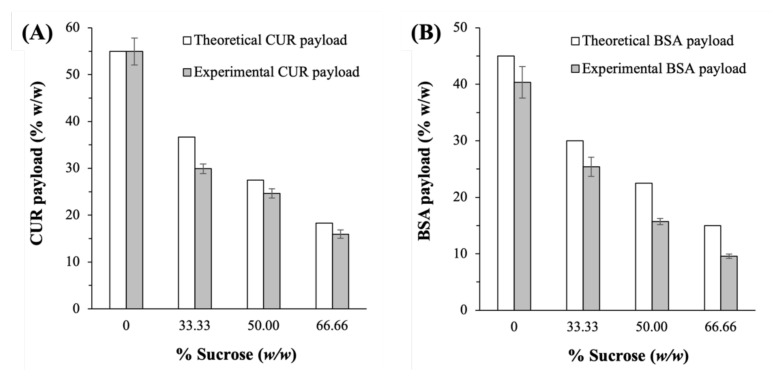
Experimental vs. theoretical payloads of (**A**) CUR and (**B**) BSA in the lyophilized CUR-BSA nanoplexes prepared at different sucrose mass fractions.

Before lyophilization, the CUR loading of the CUR-BSA nanoplex was determined to be equal to 54.9 ± 1.8% (*w*/*w*), with BSA making up the remaining mass (≈45%). After lyophilization in the absence of sucrose, the experimental CUR payload of the lyophilized nanoplex was found to be nearly identical to the theoretical payload (i.e., ≈55%), indicating negligible CUR loss due to lyophilization (Figure 2A). With the addition of sucrose, the experimental CUR payloads were determined to be lower than the theoretical CUR payloads by about 3 to 7%, depending on the sucrose mass fraction. Specifically, the experimental CUR payloads at 33.33%, 50.00%, and 66.66% sucrose mass fractions were equal to 29.9 ± 1.0%, 24.6 ± 0.9%, and 15.9 ± 0.9% (*w*/*w*), respectively, compared to the theoretical values of ≈36.6%, 27.5%, and 18.3%, respectively. As CUR was not known to undergo degradation upon freezing, the lower experimental CUR payloads were likely caused by the small mass loss incurred upon adding the nanoplex suspension to the sucrose solution prior to the lyophilization step. 

A similar trend was observed in the experimental BSA payloads (Figure 2B). The theoretical BSA payloads at 0, 33.33%, 50.00%, and 66.66% sucrose mass fractions were calculated to be equal to ≈45%, 30%, 22.5%, and 15%, respectively. The corresponding experimental BSA payloads were lower by between 5 and 6%, at 40.3 ± 2.8%, 25.4 ± 1.7%, 15.8 ± 0.5%, and 9.6 ± 0.4% (*w*/*w*), respectively. The similar magnitude in the deviations in the CUR and BSA experimental payloads from the theoretical values reaffirmed the postulate that the loss in payload was indeed caused by the mass loss incurred prelyophilization, instead of being caused by the thermal and mechanical stresses of lyophilization.

**Figure 3:**
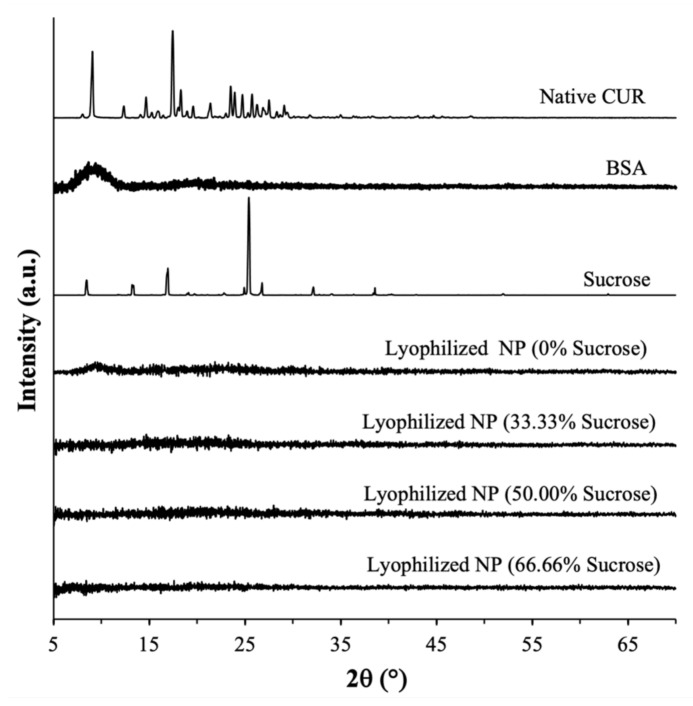
PXRD patterns of the native CUR, BSA, raw sucrose, and lyophilized CUR-BSA nanoplexes prepared at different sucrose mass fractions.

The PXRD analysis performed immediately after lyophilization showed that the CUR-BSA nanoplexes lyophilized with or without sucrose exhibited characteristics of amorphous solids, as evidenced by the absence of strong intensity peaks in their PXRD patterns. In contrast, strong intensity peaks were observed in the PXRD patterns of the native CUR (with major peaks at 2θ = 9° and 18°) and the raw sucrose (with major peaks at 2θ = 17° and 25°), indicating their native crystalline forms (Figure 3). The native BSA also exhibited amorphous solid characteristics, as reflected by the presence of amorphous halos at around 2θ = 7°–12°. The amorphicity of the lyophilized nanoplex at the three investigated sucrose mass fractions boded well for their CUR kinetic solubility enhancement capabilities, as we discuss in Section 2.2.3. Importantly, the amorphicity of the nanoplex lyophilized with sucrose signified the miscibility between sucrose and the CUR-BSA nanoplex upon lyophilization, whereby sucrose was able to form hydrogen bonds with BSA in the nanoplex and, as a result, stabilized the protein from unfolding upon the nanoplex’s exposure to external stresses [35]. In Section 2.3, the abilities of the amorphous sucrose to maintain the physicochemical properties of the CUR-BSA nanoplex upon exposure to elevated heat and humidity are examined.

### 2.2. Feasibility of Sucrose as Cryoprotectant

#### 2.2.1. Aqueous Reconstitution

The redispersion of the lyophilized CUR-BSA nanoplex in an aqueous environment should ideally lead to the recovery of the original nanoplex’s size and zeta potential, by which the intended therapeutic function of the nanoplex could be fully realized. The lyophilization of the CUR-BSA nanoplex without cryoprotectant (i.e., 0% sucrose) led to the irreversible aggregation of the nanoplex, as evidenced by $S_{f}/S_{i}$ >> 1.0, due to inter-nanoparticle fusion induced by ice crystal formation (Table 1). Despite the significantly larger size, at 1135 ± 61 nm, the zeta potential of the reconstituted nanoplex, at −27.9 ± 1.5 mV, remained comparable to the value before lyophilization, indicating the still predominant presence of BSA on the nanoplex aggregates’ surfaces. 

Adding sucrose as the cryoprotectant at 33.33% to 66.66% (*w*/*w*) mass fractions successfully prevented the irreversible nanoplex aggregation from taking place, as reflected by the $S_{f}/S_{i}$ values close to unity and the minimal change in the zeta potential (Table 1). Moreover, the relatively low PDI (<0.50) indicated that the reconstituted nanoplexes exhibited good monodispersity in their sizes. The good aqueous reconstitution was attributed to sucrose, which occupied the interstitial spaces among the nanoplexes to prevent their aggregation. Upon dispersion in an aqueous environment, the sucrose readily dissolved and, in turn, liberated the nanoplex. The ability of sucrose to prevent the aggregation of the nanoplex after lyophilization reported in the present work was consistent with previous studies that reported the ability of sucrose to prevent the aggregation of other therapeutic nanoparticles (e.g., solid lipids, polymers, and liposomes) [28,29]. 

#### 2.2.2. CUR Dissolution Profiles 

**Figure 4:**
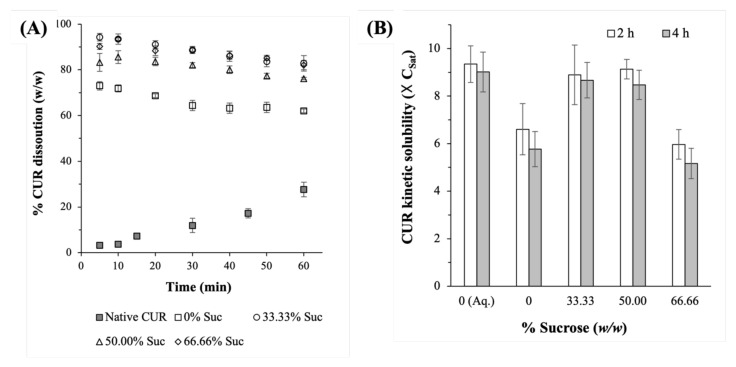
(**A**) CUR dissolution profiles and (**B**) amorphous CUR kinetic solubility of the lyophilized CUR-BSA nanoplexes prepared at different sucrose mass fractions. (“Suc” = sucrose and “Aq.” = aqueous suspension.)

The lyophilized CUR-BSA nanoplexes exhibited fast CUR dissolution rates under sink condition, with immediate release profiles where around 85–95% (*w*/*w*) of the CUR payload was dissolved after 5 min (Figure 4A). The CUR burst release rates in the first 5 min followed zero-order dissolution kinetics, with dissolution constants of approximately 14.6 min^−1^ in the absence of sucrose and around 16.6 to 18.1 min^−1^ in the presence of sucrose. For comparison, the native CUR powders exhibited less than 5% CUR dissolution after the same period. The native CUR dissolution also followed zero-order kinetics, with dissolution constant of roughly 0.43 min^−1^, denoting its significantly slower dissolution rate compared to the lyophilized nanoplex. 

On this note, the percentage dissolved CUR began to slowly decrease after peaking at 10 min due to the well-known hydrolytic degradation of CUR in PBS at physiological pH [36]. The fast dissolution characteristics of the CUR-BSA nanoplex, which were attributed to the nanoplex’s metastable amorphous form and nanoscale size, were thus preserved after lyophilization. This could be attributed to the effective aqueous reconstitution of the lyophilized nanoplex using sucrose as a cryoprotectant. 

The effects of the sucrose mass fraction on the CUR dissolution rates were found to be minimal, as evidenced by the small variations in the zero-order dissolution constants, i.e., ≈17.8, 16.6, and 18.1 min^−1^ at sucrose mass fractions of 33.33%, 50.00%, and 66.66%, respectively. The comparable dissolution rates at different sucrose mass fractions were not unexpected considering that the lyophilized nanoplexes exhibited nearly identical aqueous reconstitutions for the three investigated sucrose mass fractions (Table 1). 

In the absence of sucrose, however, a slower CUR dissolution rate was observed, where roughly 70% (*w*/*w*) of the CUR payload was dissolved after 10 min. The slower CUR dissolution was caused by the reduced surface areas available for dissolution due to the aforementioned poor aqueous reconstitution of the lyophilized nanoplex in the absence of sucrose. When the lyophilized nanoplex was not fully reconstituted to an individual nanoplex, the surface area available for dissolution was reduced, as the surface area was inversely proportional to the particle diameter [37]. 

#### 2.2.3. Amorphous CUR Kinetic Solubility

A useful indicator for the preservation of the CUR-BSA nanoplex’s physicochemical properties after lyophilization was its amorphous CUR kinetic solubility enhancement capability. The lyophilized nanoplexes prepared at 33.33% and 50.00% sucrose mass fractions were found to exhibit high CUR kinetic solubilities, at approximately 8.9–9.1 × C_Sat_. The high kinetic solubility was maintained over 4 h, which should provide ample time for the absorption of the dissolved CUR upon in vivo translation (Figure 4B). It is worth mentioning that the CUR kinetic solubility would eventually decrease to the thermodynamically stable value (i.e., C_Sat_) after about 12 h (data not shown) due to the precipitation of the supersaturated CUR solution. Notably, under the same experimental conditions, the CUR kinetic solubility exhibited by the lyophilized nanoplexes prepared at 33.33% and 50.00% sucrose mass fractions were comparable in magnitude to that exhibited by the aqueous CUR-BSA nanoplex suspension (“0 (Aq.)”). Their similarities signified the nearly negligible impact of lyophilization on the amorphous CUR kinetic solubility. 

On the other hand, a lower CUR kinetic solubility at 5.1–5.8 × C_Sat_ was observed for the lyophilized nanoplex prepared at a 66.66% sucrose mass fraction, suggesting that the presence of excessive sucrose in the dissolution medium reduced the CUR kinetic solubility. Likewise, the CUR kinetic solubility was also lower, at around 5.8–6.6 × C_Sat_, for the nanoplex lyophilized without sucrose. The lower CUR kinetic solubility in the absence of sucrose was postulated to be caused by the abovementioned slower dissolution rate of the lyophilized nanoplex without sucrose due to its poor aqueous reconstitution. The slower dissolution rate, in turn, increased the solution-mediated crystallization tendency of the amorphous solids during dissolution, resulting in a partial loss of kinetic solubility [38]. 

### 2.3. Physicochemical Stability after 30-Day Exposures to 40 °C and 75% RH

#### 2.3.1. Amorphous Form

**Figure 5:**
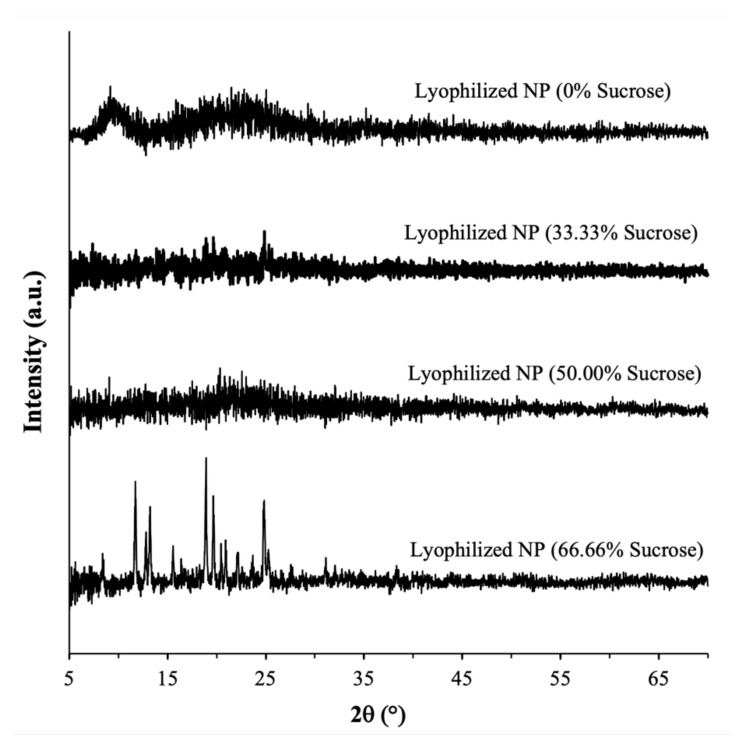
PXRD patterns of the lyophilized CUR-BSA nanoplexes prepared at different sucrose mass fractions after 30-day exposures at 40 °C and 75% RH.

A PXRD analysis performed after the 30-day exposures showed that the lyophilized CUR-BSA nanoplexes prepared at 0, 33.33%, and 50.00% sucrose mass fractions remained amorphous after prolonged exposures to elevated heat and humidity (Figure 5), signifying their high amorphous form stability. The amorphous form stability of the CUR-BSA nanoplex lyophilized without sucrose indicated that the strong electrostatic and hydrophobic bindings between the CUR and BSA molecules were sufficient to suppress the molecular mobility of the amorphous CUR and BSA during the exposures, preventing their recrystallization. Even though the CUR-BSA nanoplex did not recrystallize after the exposures, as we show in Section 2.3.4, the inclusion of sucrose was needed in order to maintain the physicochemical stability of BSA in the nanoplex upon the nanoplex’s exposure to elevated heat and humidity. 

On the other hand, a PXRD analysis of the lyophilized nanoplex prepared at a 66.66% sucrose mass fraction showed several strong intensity peaks at 2θ ≈ 12°, 13°, 18°, 20°, and 25, indicating that an amorphous-to-crystalline transformation was taking place. As the nanoplex lyophilized without sucrose was not found to recrystallize postexposure, denoting the inherently high stability of the CUR-BSA nanoplex, the amorphous-to-crystalline transformation observed at a 66.66% sucrose mass fraction was postulated to be triggered by the recrystallization of amorphous sucrose. 

In this regard, pure amorphous sucrose prepared by lyophilization was known to possess a strong recrystallization propensity [39,40]. Sucrose recrystallization in the lyophilized nanoplex prepared at a 66% sucrose mass fraction was evident from the appearance of a strong-intensity peak at 2θ ≈ 25°, which was uniquely present in the PXRD pattern of the raw sucrose shown in Figure 3. The sucrose recrystallization caused the nanoplex to be immiscible with sucrose, which in turn destabilized the nanoplex. As a result, the CUR in the nanoplex also underwent an amorphous-to-crystalline transformation, as evidenced by the appearance of strong-intensity peaks at 2θ ≈ 12° and 18°, which were present in the PXRD pattern of the native CUR shown in Figure 3. 

In short, the PXRD analysis performed postexposure showed that a high sucrose mass fraction of 66.66% led to the recrystallization of both the sucrose and the lyophilized nanoplex. The recrystallization, nevertheless, had no adverse impacts on the other characteristics of the lyophilized nanoplex (e.g., aqueous reconstitution, CUR dissolution, payloads, and BSA’s stability), as demonstrated in the next sections. 

#### 2.3.2. Aqueous Reconstitution 

The CUR-BSA nanoplexes lyophilized with sucrose maintained good aqueous reconstitution postexposure, as evidenced by their $S_{f}/S_{i}$ values close to unity (Table 2). In fact, there were statistically insignificant variations in the $S_{f}/S_{i}$ and zeta potential values before and after the 30-day exposures. Thus, the BSA in the nanoplex was able to maintain its colloidal stabilizing role after the exposures despite its diminishing structural integrity, as discussed in Section 2.3.4. Nevertheless, the PDIs of the reconstituted nanoplexes postexposure were higher, at ≈0.6–0.7, compared to the values before the exposures (≈0.25–0.35), as reported earlier in Table 1. The higher PDIs suggested a wider size distribution of the reconstituted nanoplexes postexposure, likely caused by the incomplete reconstitution of a small fraction of the lyophilized nanoplexes. 

#### 2.3.3. Payloads and Dissolution Profiles

**Figure 6:**
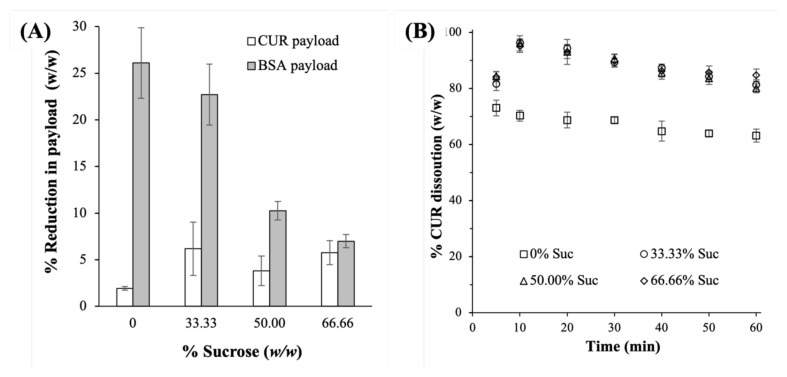
(**A**) Reduction in the CUR and BSA payloads and (**B**) CUR dissolution profiles of the lyophilized CUR-BSA nanoplexes after 30-day exposures at 40 °C and 75% RH.

As both CUR and BSA solids were known to be prone to chemical degradation upon their exposures to elevated heat and humidity [32,41], the ability of sucrose to maintain the chemical stability of the lyophilized CUR-BSA nanoplex was investigated in terms of the percentage reductions in the CUR and BSA payloads after the 30-day exposures. The results showed that the CUR payload was minimally reduced (≤6%) postexposure, independent of the sucrose mass fractions (Figure 6A). In contrast, the percentage reductions in the BSA payload postexposure were significantly higher, in the range of 7% to 26%, depending on the sucrose mass fraction. Notably, the percentage reductions in the BSA payload decreased with increasing sucrose mass fractions, thereby indicating the protective effects of sucrose. Specifically, the BSA payloads were reduced by 26.1 ± 3.7%, 22.7 ± 3.2%, 10.2 ± 1.0%, and 7.0 ± 0.7% at 0, 33.33%, 50.00%, and 66.66% sucrose mass fractions, respectively. The observed decreases in the BSA payloads postexposure were consistent with the observed increased protein aggregation postexposure, as discussed in Section 2.3.4.

The larger reductions in the BSA payloads observed in the lyophilized CUR-BSA nanoplexes prepared at lower sucrose mass fractions, however, were not found to have any significant impacts on their CUR dissolution profiles. The burst release profiles observed for the lyophilized nanoplexes postexposure remained independent of their sucrose mass fractions (Figure 6B). Thus, the effects of the sucrose mass fraction on CUR dissolution were found to be minimal before and after the exposures. The slower CUR dissolution rate of the nanoplex lyophilized without sucrose was also observed postexposure, which was not unexpected considering that its aqueous reconstitution remained poor after exposure. 

#### 2.3.4. Protein Structural Integrity 

**Figure 7:**
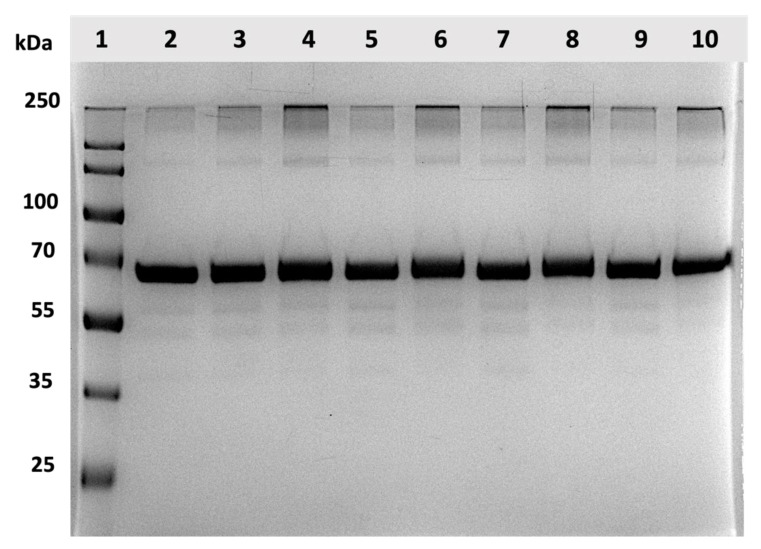
SDS-PAGE gel images of the lyophilized CUR-BSA nanoplexes before and after 30-day exposures at 40 °C and 75% RH. Column 1 is the protein ladder; Column 2 is the raw BSA; Columns 3, 5, 7, and 9 are pre-exposure lyophilized nanoplexes prepared at 0, 33.33%, 50.00%, and 66.66% sucrose mass fractions, respectively; Columns 4, 6, 8, 10 are postexposure lyophilized nanoplexes prepared at 0, 33.33%, 50.00%, and 66.66% sucrose mass fractions, respectively.

In this section, the impacts of lyophilization on the structural integrity of the BSA in the CUR-BSA nanoplex were investigated to examine the cryoprotective effects of sucrose. Subsequently, the BSA’s integrity in the lyophilized nanoplex after the 30-day exposures of elevated heat and humidity was investigated. As a benchmark, the SDS-PAGE analysis showed that the raw BSA (Column 2) exhibited a monomer protein band with MW ≈ 66 kDa and low-intensity oligomers at MW ≈ 250 kDa, in agreement with the previous SDS-PAGE results of BSA [42] (Figure 7). 

**Figure 8:**
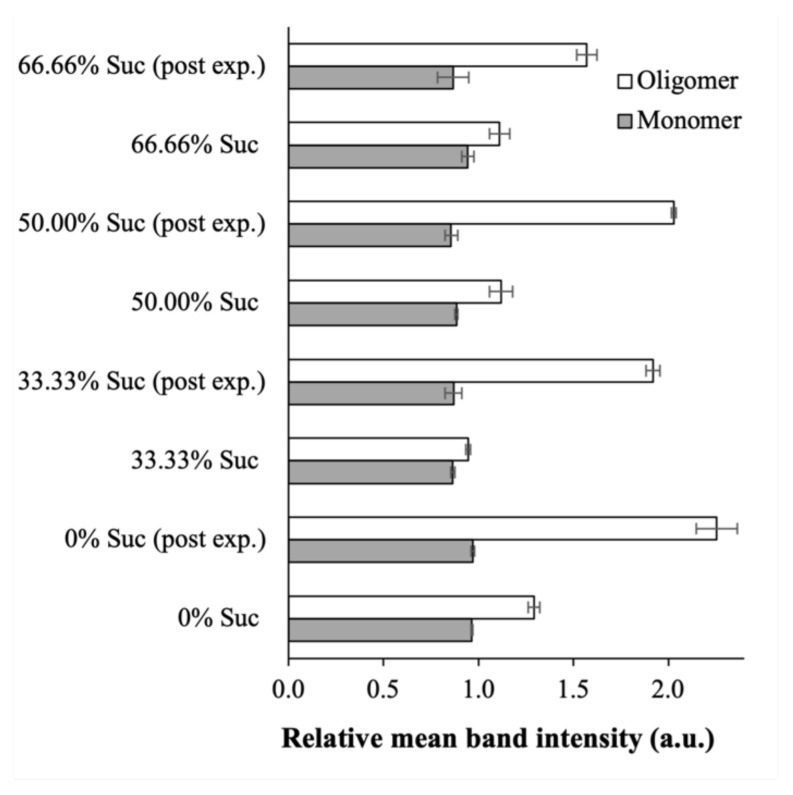
Relative mean intensities of the protein bands of the lyophilized nanoplexes prepared at different sucrose mass fractions before and after the 30-day exposures. The relative mean intensities were calculated with respect to the band intensity of the raw BSA (“post exp.”: postexposure).

The SDS-PAGE gel images of the lyophilized nanoplexes prepared at different sucrose mass fractions (Columns 5, 7, and 9) showed similar protein bands as the raw BSA. No obvious increase in the intensity of the oligomer bands was observed. This signified an absence of significant protein aggregation due to unfolded proteins after lyophilization. Using sucrose as the cryoprotectant, the densitometry analysis revealed that the relative mean intensities of the protein bands (both monomer and oligomer) of the lyophilized nanoplexes were either equal or slightly smaller/larger than unity when calculated with respect to the raw BSA’s intensities (Figure 8). In contrast, the oligomer band’s relative intensity in the nanoplex lyophilized without sucrose (Column 3) was equal to ≈1.3, denoting a roughly 30% increase due to protein aggregation. In short, the structural integrity of the BSA in the nanoplex was maintained after lyophilization with sucrose, signifying the good cryoprotective activity of sucrose. Sucrose’s good cryoprotective activity towards albumin reported in the present work was in agreement with the results of previous studies [32,43]. 

On the other hand, the gel images of the lyophilized nanoplexes after the 30-day exposures (Columns 4, 6, 8, and 10) showed increased relative intensities in the oligomer protein bands compared to the pre-exposure values, while the monomer bands’ relative intensities were minimally affected (Figure 7). Notably, the densitometry analysis showed that the increase in the oligomer band’s relative intensities was dependent on the sucrose mass fractions (Figure 8). For the CUR-BSA nanoplex lyophilized without sucrose (Column 4), the relative mean intensity of the oligomer band postexposure was equal to 2.26 ± 0.11 times that of the raw BSA, indicating that significant protein aggregation was taking place. 

The relative oligomer band intensities of the lyophilized nanoplexes prepared at 33.33%, 50.00%, and 66.66% sucrose mass fractions (Columns 6, 8, and 10, respectively) were lower, at 1.92 ± 0.04, 2.03 ± 0.01, and 1.57 ± 0.05, respectively. These results signify the ability of sucrose to help stabilize the BSA in the lyophilized nanoplex upon the nanoplex’s exposure to elevated heat and humidity, which could be attributed to hydrogen bond interactions between sucrose’s abundant hydroxyl groups and the amino groups of BSA. Nevertheless, a significant sucrose mass fraction (>50.00%) in the lyophilized products was needed to maintain the BSA’s structural integrity postexposure. 

## 3. Materials and Methods 

### 3.1. Materials

Curcumin (CUR) (≥95 wt.% curcuminoid) was purchased from Alfa Aesar (Singapore, Singapore). Bovine serum albumin (BSA) with a molecular weight (MW) of 66.5 kDa, potassium hydroxide (KOH), glacial acetic acid (AA), phosphate-buffered saline (PBS, pH 6.8), sodium dodecyl sulfate (SDS), ethanol, acetonitrile, trifluoroacetic acid (TFA), and sodium chloride (NaCl) were purchased from Sigma Aldrich (Singapore, Singapore). Sucrose (≥99% wt.%) was purchased from Tokyo Chemical Industry (Tokyo, Japan). In addition, 30% Acrylamide/Bis-acrylamide (29:1 *w*/*w*), tris, sodium dodecyl sulfate, ammonium persulfate, *N*,*N*,*N*′,*N*′-Tetramethyl ethylenediamine (TEMED), ß-mercaptoethanol, Coomassie Brilliant Blue G-250, and an SDS-PAGE sample loading buffer were purchased from Bio-Rad (Singapore, Singapore). A PageRuler™ prestained protein ladder (15 to 250 kDa) was purchased from Thermo Scientific (Singapore, Singapore).

### 3.2. Methods

#### 3.2.1. Preparation of CUR-BSA Nanoplex

Anionic CUR solution was prepared by dissolving CUR with pK_a_ values of 7.8, 8.5, and 9.0 [44] in 0.1M KOH (pH 13) at 5 mg/mL. Separately, a cationic BSA solution was prepared by dissolving BSA with an isoelectric point (pI) of 4.5–5.0 [45] in a 0.6% (*v*/*v*) aqueous AA solution (pH 2.9) at 5 mg/mL. Equal volumes (5 mL) of the CUR and BSA solutions were mixed immediately after their preparation to minimize the alkaline degradation of CUR. The mixed solution was vortexed for 10 s and equilibrated for 5 min. The electrostatic complexation took place between the negatively charged phenolic group of CUR at a pH above its pKa and the positively charged lysine residues of BSA at a pH below its pI [46]. 

Afterwards, the resultant CUR-BSA nanoplex suspension underwent two cycles of centrifugation at 14,000× *g* for 10 min and redispersion in deionized water to remove excess CUR and BSA that did not form the nanoplex. The CUR loading in the nanoplex was determined in triplicate by dissolving a known mass of the nanoplex in an 80% (*v*/*v*) aqueous ethanol solution, after which the CUR concentration in the ethanol solution was determined by UV-Vis spectroscopy (UV Mini-1240, Shimadzu, Japan) at an absorbance wavelength of 423 nm [25]. The BSA loading in the nanoplex was subsequently calculated from the CUR loading. 

#### 3.2.2. Lyophilization of CUR-BSA Nanoplex 

The washed CUR-BSA nanoplex suspension was concentrated to approximately 5.3 mg/mL, after which it was added to an aqueous sucrose solution of the same concentration (i.e., 5.3 mg/mL) at different volume ratios. Three sucrose mass fractions were investigated, i.e., 33.33%, 50.00%, and 66.66%, corresponding to nanoplex-to-sucrose mass ratios of 2:1, 1:1, and 1:2, respectively. The resultant CUR-BSA nanoplex suspension in sucrose was lyophilized for 24 h in an Alpha 1-2 LDPlus freeze dryer (Martin Christ, Germany) at −52 °C and 0.05 mbar. A nanoplex lyophilized without sucrose was used as a control. The lyophilized nanoplex powders were stored in a dry cabinet prior to their characterizations. The morphology of the lyophilized nanoplex was examined by field emission scanning electron microscopy (FESEM) using a JSM 6700F microscope (JEOL, Boston, MA, USA). 

#### 3.2.3. Aqueous Reconstitution

The aqueous reconstitution of the lyophilized CUR-BSA nanoplex was investigated following the protocols presented in Yu et al. [47] with slight modifications. Briefly, the lyophilized nanoplex powders were redispersed using a pipette tip in deionized water at 0.1 mg/mL for 10 min without stirring. The number-averaged size of the reconstituted nanoplex ($S_{f}$) was measured after a 100-fold dilution by dynamic light scattering (DLS) using a Brookhaven 90 Plus Nanoparticle Size Analyzer (Brookhaven Instruments Corporation, USA). The aqueous reconstitution was characterized from six replicates by the ratio of $S_{f}$ to $S_{i}$, where $S_{i}$ represented the initial number-averaged size of the CUR-BSA nanoplex before lyophilization. Good aqueous reconstitutions were typically characterized by $S_{f}/S_{i}$ values less than 1.5 [47]. The polydispersity index (PDI) and zeta potential of the reconstituted nanoplex were also characterized by DLS [48,49]. 

#### 3.2.4. Experimental CUR and BSA Payloads

The experimental CUR (or BSA) payload was defined as the mass of CUR (or BSA) contained per unit of mass of the lyophilized CUR-BSA nanoplex + sucrose. The drug payload has a significant influence on the dissolution rate and the physicochemical stability of pharmaceutical products [50]. The payload was characterized in triplicate by dispersing a known mass of the lyophilized powders (5 mg) in deionized water to dissolve the sucrose. The remaining particulate suspension containing the CUR-BSA nanoplex was then centrifuged at 14,000× *g* for 5 min to remove the sucrose in the supernatant. Next, the sedimented pellets were dissolved in a 50% (*v*/*v*) aqueous ethanol solution to dissolve CUR and simultaneously release BSA. The CUR and BSA payloads were determined simultaneously by high-performance liquid chromatography (HPLC) at a detection wavelength of 220 nm [51] in an Agilent 1100 HPLC system (Agilent Technologies, USA). The HPLC analysis was performed using a ZORBAX Eclipse Plus C18 column (250 Å~4.6 mm, 5 μm particle size) with acetonitrile/water/TFA (60:40:0.05 *v*/*v*/*v*) as the mobile phase at flowrate of 1.0 mL/min. In this condition, the CUR and BSA retention times were approximately 5.8 min and 1.7 min, respectively. The BSA retention time was in agreement with the HPLC results of BSA reported by Boiero et al. [52]. The experimental CUR and BSA payloads were compared with their theoretical payloads, which were calculated by assuming zero mass loss. 

#### 3.2.5. CUR Dissolution Profiles

The CUR dissolution from the lyophilized CUR-BSA nanoplex under sink condition was characterized in triplicate. Herein, the sink condition was defined as a condition in which the maximum concentration of the solute (i.e., CUR) in the dissolution medium was equal to ¼ of its thermodynamic saturation solubility (C_Sat_). PBS (pH 6.8) supplemented with 1.0% (*w*/*v*) SDS was used as the dissolution medium. The role of SDS was to increase the C_Sat_ of CUR to roughly 0.12 mg/mL to facilitate the reliable detection of the CUR concentration by HPLC. Briefly, the lyophilized nanoplex was added at ¼ C_Sat_ to 40 mL of PBS + SDS maintained at 37 °C in a shaking incubator. Next, 1 mL aliquots were withdrawn at specific time points over 60 min, and fresh dissolution medium of the same volume was added as replenishment. The aliquots were centrifuged at 14,000× *g* for 5 min, and the CUR concentration in the supernatant was determined by HPLC as previously described.

#### 3.2.6. Amorphous CUR Kinetic Solubility

The kinetic solubility of the amorphous CUR in the lyophilized CUR-BSA nanoplex was characterized in six replicates by adding the lyophilized powders in excess at 10 × C_Sat_ to 15 mL of PBS (pH 6.8) maintained at 37 °C in a shaking incubator. The C_Sat_ value of CUR in PBS (pH 6.8) without SDS was equal to approximately 4.15 µg/mL. Two aliquots were withdrawn after 2 h and 4 h and centrifuged at 14,000× *g* for 5 min. The CUR concentration in the supernatant was determined by HPLC as previously described. The CUR kinetic solubility was reported in terms of its ratio, calculated with respect to C_Sat_. For comparison, the kinetic solubility of the CUR-BSA nanoplex suspension was also characterized under the same experimental conditions. 

#### 3.2.7. Amorphous Form Stability

The lyophilized CUR-BSA nanoplex was stored in a desiccator set to 40 ^°^C and 75% relative humidity (RH) for 30 days. The 75% relative humidity condition was generated by placing an open container of a saturated NaCl solution in the desiccator overnight at 40 °C. The amorphous forms of the lyophilized nanoplex were characterized before and after the 30-day exposures by powder X-ray diffraction (PXRD) using a D8 Advance X-ray Diffractometer (Bruker, Germany) performed between 10° and 70° (2*θ*) with a step size of 0.02° and a scanning rate of 1.2°/min. PXRD analyses of the native CUR, native BSA, and raw sucrose were also performed. The PXRD characterization protocols were in agreement with the protocols presented in [53,54,55]. 

#### 3.2.8. Protein Structural Integrity

The structural integrity of BSA in the lyophilized CUR-BSA nanoplex before and after the 30-day exposure to 40 °C and 75% RH was examined by an SDS-PAGE analysis using four replicates. The SDS-PAGE was performed following the protocols of Liu and Jing [56]. The SDS-PAGE gel was prepared with 10% (*w*/*v*) acrylamide resolving gel and 5% (*w*/*v*) acrylamide stacking gel. Briefly, 2 mg of the lyophilized nanoplex powders were dispersed in 2 mL of deionized water. The resulting suspension was then mixed with the loading buffer containing 5% (*v*/*v*) ß-mercaptoethanol and heated at 100 °C for 5 min. Next, 30 µL of the suspension containing roughly 2 ng of BSA was loaded into the SDS-PAGE gel. The gel electrophoresis was run at 110 V for 2 h. 

Afterwards, the gel was stained with Coomassie Brilliant Blue G-250 for 1 h and was subsequently destained in deionized water for 1 h. Images of the stained protein bands were visualized and taken by a gel documentation system (GeneSys, Frederick, MD, USA). A control run was performed for the native BSA in which the protein ladder was used to estimate the molecular weight. The structural integrity of BSA before and after the 30-day exposure was characterized in terms of the relative mean intensity of the protein bands with respect to the native BSA’s bands. The mean band intensity was quantified by a densitometry analysis using ImageJ software (NIH, Bethesda, MD, USA). 

## 4. Conclusions

The nanoplex lyophilized with sucrose exhibited good aqueous reconstitution, with the reconstituted nanoplex having a size and zeta potential comparable to the nanoplex suspension. The good aqueous reconstitution enabled the lyophilized nanoplex to maintain the fast CUR dissolution rates and high amorphous CUR kinetic solubility exhibited by the nanoplex suspension. The structural integrity of BSA in the nanoplex was not adversely affected by lyophilization. The effects of the sucrose mass fraction on the aqueous reconstitution, CUR dissolution profiles, and BSA’s structural integrity after lyophilization were found to be insignificant. Nevertheless, the sucrose mass fraction played an important role in maintaining the physicochemical stability of the BSA in the CUR-BSA nanoplex upon its exposure to elevated heat and humidity. The percentage reduction in the BSA payload and the degree of protein aggregation after the exposure were found to be lower at higher sucrose mass fractions. Despite the diminished BSA integrity postexposure, the good aqueous reconstitution, high CUR payload, fast CUR dissolution rates, and amorphous forms of the lyophilized nanoplex remained evident. In short, sucrose possessed cryoprotective and physicochemical stabilizing effects for the CUR-BSA nanoplex. 

## Figures and Tables

**Table 1:** Aqueous reconstitution of the lyophilized CUR-BSA nanoplex (NP).

% Sucrose (*w*/*w*)	Reconstituted NP size $S_{f}(\mathrm{nm})$	PDI	Zeta potential (mV)	$S_{f}/S_{i}$
0	1135 ± 61	0.55 ± 0.08	−27.9 ± 1.5	10.40 ± 0.61
33.33	100 ± 17	0.26 ± 0.03	−24.8 ± 1.1	0.92 ± 0.11
50.00	103 ± 7	0.34 ± 0.02	−24.6 ± 0.9	0.94 ± 0.06
66.67	99 ± 7	0.35 ± 0.02	−25.1 ± 1.1	0.91 ± 0.06

**Table 2:** Aqueous reconstitution of the lyophilized CUR-BSA nanoplexes prepared at different sucrose mass fractions after 30-day exposures at 40 °C and 75% RH.

% Sucrose (*w*/*w*)	Reconstituted NP Size $S_{f}(\mathrm{nm})$	PDI	Zeta Potential (mV)	$S_{f}/S_{i}$
0	1523 ± 99	0.70 ± 0.08	−25.5 ± 0.9	12.52 ± 1.02
33.33	124 ± 34	0.70 ± 0.07	−22.9 ± 1.8	1.13 ± 0.22
50.00	98 ± 37	0.61 ± 0.18	−21.4 ± 0.9	0.90 ± 0.16
66.67	119 ± 36	0.68 ± 0.03	25.6 ± 1.2	1.09 ± 0.24

## Data Availability

Data available upon request.

## Abbreviations

AA	Acetic acid
a.u.	Arbitrary unit
BSA	Bovine serum albumin
C_Sat_	Thermodynamic saturation solubility
CUR	Curcumin
DLS	Dynamic light scattering
FESEM	Field emission scanning electron microscope
HPLC	High-performance liquid chromatography
MW	Molecular weight
NP	Nanoplex
PBS	Phosphate-buffered saline
PDI	Polydispersity index
pI	Isoelectric point
PXRD	Powder X-ray diffraction
RH	Relative humidity
SDS-PAGE	Sodium dodecyl sulfate–polyacrylamide gel electrophoresis
$S_{f}$	Nanoplex size after reconstitution
$S_{i}$	Original nanoplex size before lyophilization
Suc	Sucrose
UV-Vis	Ultraviolet-visible
